# Considerations about the implementation of an autism screening program in Iran from the viewpoints of professionals and parents: a qualitative study

**DOI:** 10.1186/s12888-021-03061-0

**Published:** 2021-01-23

**Authors:** Hassan Shahrokhi, Akbar Ghiasi, Kamal Gholipour, Leila Mehdizadeh Fanid, Hamid Reza Shamekhi, Shabnam Iezadi

**Affiliations:** 1 Research Center of Psychiatry and Behavioral Science, Tabriz University of Medical Sciences, Tabriz, Iran; 2 HEB School of Business & Administration, University of the Incarnate Word, San Antonio, TX USA; 3 Tabriz Health Services Management Research Center, School of Management and Medical Informatics, Tabriz University of Medical Sciences, Tabriz, Iran; 4 Division of Cognitive Neuroscience. Department of Psychology, Faculty of Education and Psychology. University of Tabriz, Tabriz, Iran; 5 Education Development Office (EDO), Faculty of Medicine, Tabriz Azad Islamic University, Tabriz, Iran; 6 Hospital Management Research Center, Iran University of Medical Sciences, Tehran, Iran

**Keywords:** Autism Spectrum disorders, ASD, Screen, Qualitative study, Challenges

## Abstract

**Background:**

The aims of this study were to explore to explore the viewpoints of parents of children with Autism Spectrum Disorders (ASD) and professionals regarding the implementation of screening programs for ASD, to explore the challenges of the implementation of a universal screening program for ASD in Iran from their viewpoints, and, to explore their recommendations to overcome the potential challenges.

**Method:**

This qualitative study was conducted using an inductive content analysis, between June 2018 and December 2018, in East-Azerbaijan province of Iran. Data was collected through in-depth interviews and focus group discussions. The participants were purposively selected among two groups: representatives of health system and representatives of children with ASD. A sample of 32 parents and 30 professionals were recruited in this study.

**Results:**

Totally, 9 main themes and 23 sub-themes were extracted in three main areas including: viewpoints of the participants about universal screening for ASD, challenges in implementation of the universal screening program, and participants’ recommendations about how to overcome the potential challenges. Main challenges in implementation of the universal screening program included: shortages of ASD screening tools, weakness of the health system, lack of coordination among the ASD service providers, and social and ethical issues.

**Conclusion:**

The parents and the professionals had different viewpoints about the implementation of ASD universal screening program in Iran. According to the professionals, there is not enough rational to implement ASD screening program for all children. However, the parents believed that universal screening program is inevitable, and it should be implemented in primary health centers during the early child-care visits. The results of this study open up unspoken issues that could help in initiating the screening program not only in Iran but also in other low- and middle-income countries as well.

**Supplementary Information:**

The online version contains supplementary material available at 10.1186/s12888-021-03061-0.

## Background

Autism Spectrum Disorders (ASD) are behaviorally defined neurodevelopmental disorders such as a wide range of possible developmental impairments in reciprocal social interaction or communication, and stereotyped, repetitive, or limited behavioral repertoire [1]. According to the World Health Organization (WHO), about one in every 160 children has an ASD worldwide, which accounts for 0.3% of the global burden of disease. The prevalence of ASD differs across countries. However, the prevalence of ASD is relatively unknown in most of the low- and middle-income countries (LMICs) [2]. A better understanding of the potential for autism screening programs in LMICs is warranted given the global imbalances in population prevalence and knowledge on autism [3]. According to the World Bank statistics in 2017, more than 61% of all children in the world live in LMICs [4]. Nevertheless, results of a systematic review by Elsabbagh et al. (2012) showed that most of the studies on the prevalence of ASD were from high-income countries, few of them were from middle-income countries (only one study from Iran), and none of them was from a low-income country [1]; on the other hand, there is a major disparity in access to a comprehensive range of services for children with ASD [3]. According to the study of Samadi et al. in Iran (2012), the prevalence of ASD was 6.26 per 10,000. In this study, 1.3 million five-year-old children were screened for autism over three academic years [5].

Although children with ASD experience the same problems as do other children, they may suffer additional problems like several comorbid conditions. They are more vulnerable to inadequate access to supportive activities (e.g. social, financial, and emotional supports), education, health care, and chronic non-communicable diseases [2]. In addition, children with ASD are at greater risk of violence, injury, and abuse [6–8]. Several studies have shown that early diagnosis of ASD followed by early intervention may reduce the undesirable consequences of the disorder [9, 10]. Implementation of an ASD screening program might increase the likelihood of early diagnosis of the disorder and may result in early provision of services for the affected children. Nonetheless, other studies concluded that the early diagnosis of the disorder does not guarantee the early provision of services [11, 12]. Therefore, there is no consensus among professionals regarding the implementation of ASD screening program for all children. Although empirical evidence indicates that ASD can be diagnosed during the first two years of life [13], early diagnosis has not become the usual practice in LMICs yet. There are numerous obstacles related to the early diagnosis including limited diagnostic resources, lack of follow-up in screening programs, and cultural barriers like social stigma [9]. In reality, in Iran, the average age of the diagnosis of ASD is approximately 5–6 years since birthday [14]. Such delay in diagnosis could be more prevalent in low-socioeconomic status population [15, 16].

In Iran, routine child-care visits are provided for children in primary health centers in the first six years of life and include vaccination and child growth monitoring. General developmental screening is conducted using Ages and Stages Questionnaire (ASQ) at each child-care visit [17]. However, in Iran, detection of children with ASD or other similar conditions is officially done through the pre-school medical examination that is done prior to the registration for the first grade of primary school (at age six), which means a major delay in diagnosis [5]. A universal screening program for children at specific ages (e.g. children of 3-year-old) provided in primary health centers as a part of the routine child-care visits seems to be a promising strategy for early diagnosis of ASD regardless of the peoples’ socio-economic status [9].

There is a controversial argument among policymakers about the implementation of ASD universal screening program [9, 18]. In 2016, the US Preventive Services Task Force (USPSTF) provided recommendations for the universal screening program. The USPSTF did not have enough evidence on the benefits of screening for ASD in children for whom no signs of ASD have been detected [19]. The American Academy of Pediatrics, on the other hand, has a different view on general screening for both developmental delays and ASD. The American Academy of Pediatrics has recommended screening for all 18- to 24-month-old children during regular well-child doctor visits [20].

In recent years, a growing concern has been seen among policymakers regarding the universal screening program for ASD in Iran. The aim of the implementation of the universal screening program is to improve the children’s developmental screening process and to speed up the diagnosis of ASD. However, screening for behavioral and psychosocial problems among young children is challenging, because the early identification of cases should be followed by further health evaluation and treatment and supportive interventions for both children and their families [20]. On the other hand, there is no knowledge on if it is appropriate to implement the universal screening for ASD in the current situation in Iran. Moreover, there are little empirical studies about the opinions of the parents of children with ASD and professionals regarding the challenges and requirements of the implementation of ASD screening program in Iran [3]. Exploring the importance and challenges of ASD screening in Iran from the viewpoints of ASD stakeholders may improve the decision-making process, and also, would lead to identifying any fundamental problems regarding the screening program before its actual implementation. In this study, we have three main aims including 1) to explore the viewpoints of parents of children with ASD and professionals regarding the implementation of screening programs for ASD, 2) to explore the challenges of the implementation of a universal screening program for ASD in Iran from their viewpoints, and, 3) to explore their recommendations to overcome the potential challenges.

## Methods

### Design

**Fig. 1:**
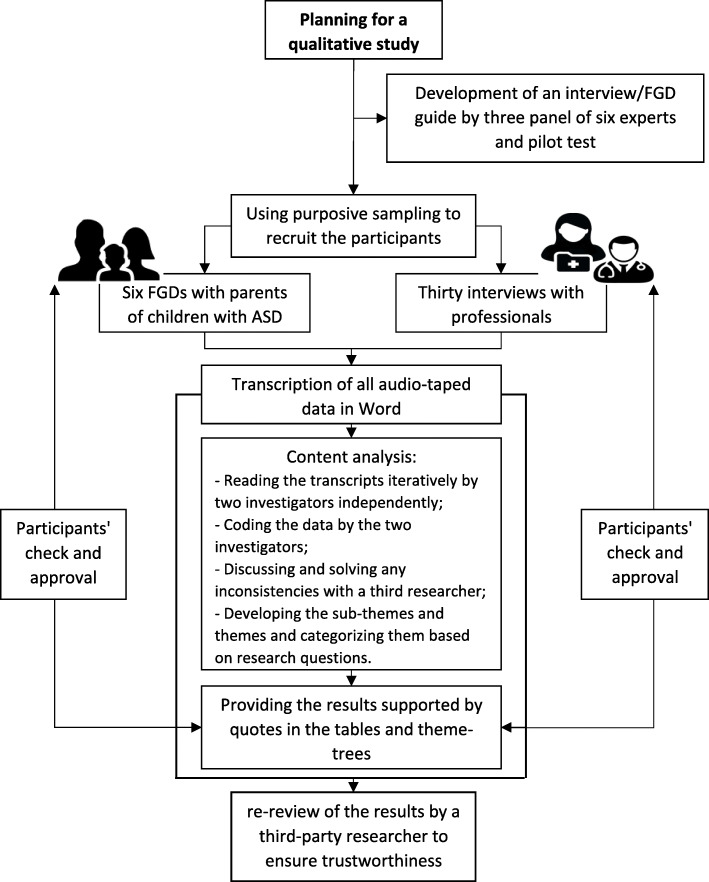
a flow diagram of the study process

Screening for autism is a context-specific program and faces major implementation challenges [21], therefore, needs special consideration when planning for the implementation of this program. Caregivers of children with ASD (especially their parents) and professionals in the field of ASD have a deep understanding of the challenges that could influence the universal screening for ASD and were considered as key informants. Therefore, a qualitative study with phenomenology approach and using content analysis was conducted between June 2018 and December 2018, in East-Azerbaijan province of Iran, to address three main questions: how do caregivers of children with ASD and professionals perceive the importance of screening children for ASD?; What are the potential challenges of universal screening of children for ASD?; and, what are their suggestions/recommendations to overcome the potential challenges? We used Merriam & Tisdell’s practical guide to direct our study [22]. A flow diagram which summarizes the process of this study is presented in Fig. 1.

### Context

East-Azerbaijan province is located in the North-West of Iran. The local language of the people in East-Azerbaijan is Azeri Turkish. Like the other regions of Iran, government provides family- and child-health care in primary health centers. However, ASD services are not provided in this system. Neither public nor private insurance cover ASD rehabilitation services and they only cover small portion of medications [23]. Detailed information on organizational structure of services for children with ASD in East-Azerbaijan province is provided as Additional file 1.

### Participants and sampling

Purposive sampling was used to select the participants. The participants were identified among two groups: representatives of the health system and representatives of children with ASD. Inclusion criteria were different according to the types of participants (see Table 1). Representatives of the health system included two groups of participants. First, those who had a direct role in diagnosis or treatment of children with ASD such as pediatrists, therapists, psychiatrists, and psychologists. Second, those who didn’t directly provide diagnosis or treatment for children with ASD, however, they had an indirect role in providing services for these children, such as those who had the administrative experience in primary health system or disability rehabilitation organizations, or who had the experience of conducting researches in the field of ASD. Representatives of children with ASD included their close family members and mainly parents. All eligible individuals were invited to participate in the study by face-to-face meetings or telephone calls. Invitation, time, and place of the interviews were finalized one week prior to each interview/FGD by telephone calls or face-to-face meetings. One day before the interviews, a reminder was sent to each participant by a text message or a phone call.

**Table 1:** participants’ inclusion and exclusion criteria

Role	Inclusion criteria	Exclusion criteria
**Representatives of health system**	• Having more than 5 years of experiences in the field of ASD (for instance, treatment, diagnosis, research, education or, administration).	• Not interested to participate in the study
**Representatives of ASD children**	• Having a well-documented primary clinical diagnosis of ASD for their child (trough valid tools e.g. DSM-5) • Being an immediate family member or other closely involved in life of an 2–12-year-old ASD child	• Not interested to participate in the study • Inability to respond to questions

### Data collection

Data was collected through in-depth interviews and Focus Group Discussions (FGD). Data collection continued until saturation was achieved. The preliminary analysis showed that after fifth FGD and 25th interview no new codes were emerged. In order to maintain the data integrity, the same topic guides, which included the same questions, were used for both the interviews and the FGDs (it is available as Additional file 2). To achieve as much depth as possible, comprehensive open-ended questions were asked to explore participants’ viewpoints in three main areas: 1) implementation of ASD screening programs (importance, advantages, and disadvantages); 2) challenges of the implementation of ASD universal screening program in Iran; and 3) recommendations for overcoming potential challenges. Both the interviews and the FGDs were guided by an instruction developed by three panel of six experts. Participants of expert panels encompassed three child & adolescent psychiatrists, two therapists, and a psychologist. Afterward, the instruction was piloted in a small sample of six parents and three professionals (who were not included in final sample). However, no changes or withdrawal of the questions were suggested in the instruction. Whereas the parents took part in the FGDs, participants from health system were interviewed because they preferred to be interviewed in their office due to high workloads.

### FGD

In order to explore the viewpoints of the parents, six 90-min FGD sessions were held in autism rehabilitation centers, in a meeting room. In order to maintain the confidentiality of participants, none of the staff members of the autism rehabilitation centers attended the meetings, nor did they have access to the quotes. Either one of the parents of 32 children (20 mothers and 12 fathers) took part in the FGDs. A facilitator, who was a child & adolescent psychiatrist (HS) and had more than 10-year experience on ASD diagnosis and treatment, guided the sessions and asked questions. A secretary, a cognitive neuroscience specialist (LM), took notes during the FGDs and assisted the facilitator to guide the sessions. At the beginning of each FGD, every participant filled a form consisting of brief questions about their demographic characteristics including age, gender, education, etc. Informed consent was already acquired from every participant to use audio-tape. In the next step, the facilitator presented an introduction regarding ASD screening program and the aims of the study. The facilitator asked questions step-by-step and encouraged the participants to discuss each question from their experience or viewpoints. The facilitator managed the sessions in a way that every participant had a chance to discuss each question. There were no correct or false answers and all participants could express their viewpoints freely and with no hesitation. Because of the use of the tape recorder, in order to keep confidentiality, each participant got an identifier in each session, and all participants were asked to avoid calling each other by real names.

#### Interview

A total of 30 Interviews were conducted with professionals at their office. The interviews were conducted in Farsi (Persian) and each interview lasted 45 min on average. Before conducting the interview, informed consent was obtained from each interviewee for using a tape recorder. Each interviewee provided a brief demographic information about him/her at the beginning of the interview. Afterward, the interviewer (HS) reviewed the aims of the study by the interviewee and asked questions step by step and encouraged the interviewee to discuss every question freely. A secretary (LM) took note during each interview and assisted the interviewer.

At the end of each session -both the FGDs and the interviews- the facilitator checked main points, which were taken by the secretary, with all participants and corrected any miss-understanding (s). The session would end if nobody had any points or suggestions.

### Analysis

Conventional content analysis method was used for data synthesis. All the interviews and the FGDs, which had been audio-taped using a digital tape recorder, were transcribed in Word, anonymously. After transcription of all interviews and FGDs, the audio files were stored in a locked area accessible only by the first author and corresponding author. Data analysis and data collection were performed simultaneously and each interview and FGD was analyzed after its conduction. Two trained researchers (SI & AG) synthesized the collected data independently. While keeping the research questions in mind, they read transcripts word by word iteratively and coded them manually. In order to have consistency in coding, investigators compared their coded transcripts with each other, and any inconsistency was discussed by a third researcher (KG). In the next step, codes were developed into sub-themes and themes based on their semantic affinity, before placed into definitive categories [24]. In this step, theme-trees were generated in order to classify the themes and sub-themes, which were emerged from participants’ verbatims, into three main categories including participants’ viewpoints about the implementation of ASD screening programs, challenges in implementation of the universal screening program, and the participants’ recommendations to overcome the challenges. Due to the enormous qualitative data, theme-trees assisted the investigators to manage the emerged themes and classifications precisely. To avoid any prejudices, the main investigators, who conducted the analysis, had no direct role in diagnosis, treatment, rehabilitation, or other services provided for the children with ASD.

### Rigor of the study

In order to ensure the trustworthiness of the qualitative study we used triangulation. Triangulation is a strategy to increase confidence in the trustworthiness and to examine the credibility, dependability, and confirmability of the results [22]. Methodological triangulation was used to strengthen the process of qualitative inquiry by using various data collection methods including in-depth interviews and FGDs. Investigator triangulation was applied through the involvement of the research team members from various disciplines in the project from design phase to data analysis and preparing the report. Data triangulation was secured by using a wide range of raw materials, codes, concepts, and theoretical saturation. To ensure the conformability of findings and trustworthiness, the results of data synthesis as well as the main themes were presented to all participants (the parents and professionals) to be reviewed and confirmed. Furthermore, the investigators requested a third-party researcher to re-review the results and analysis process to verify the trustworthiness of the findings. Finally, the comments of peer reviewers and peer debriefing considered to improve result credibility and conformability [25, 26].

## Results

A total of 62 individuals participated in this study (see Table 2 and Table 3 for participants’ characteristics). After qualitative data synthesis, nine main themes and 23 sub-themes were extracted from the participants’ declarations. Theme trees supporting our results are available as Additional file 3. The results of this study are presented in three main sections: participants’ viewpoints about implementation of ASD screening programs, challenges of ASD universal screening program in, and participants’ recommendations to overcome the challenges. To keep participants anonymous, given that the participants were approximately homogenous in terms of age, education, degree, experience, etc., and their main distinctive feature was their main role, only the main role of the professionals were used at the end of the quotes.

**Table 2:** Characteristics of the representatives of ASD children (32 participants in the FGDs)

**Item**	**Mean**	**Standard Deviation**
Age of the participant (parent)	39.5	6.48
2003Age of child	7.5	1.9
**Item**	**Frequency**	**Percentage**
Sex of the participant		
Male	20	62.5%
Female	12	37.5%
Sex of child		
Male	25	78%
Female	7	22%
Education of the participant		
Elementary	10	31%
Diploma	10	31%
Bachelor	11	35%
Master	1	3%
Occupation of the participant		
Home-maker	15	47%
Staff	8	25%
Worker	3	10%
Self-employed	2	6%
Physician/Nurse	3	9%
Retired	1	3%

**Table 3:** Characteristics of the representatives of health system (30 participants in the interviews)

N	Role (n^**a**^)	Gender	Education	Position^**b**^	Role in ASD services
**1**	**Psychiatrist (*n*** **= 8)**	• Male (*n* = 6) • Female (*n* = 2)	• Child & Adolescent Psychiatry (*n* = 5) • Psychiatry (*n* = 3)	• Faculty member (*n* = 8) • Head of a psychiatry clinic/center (*n* = 3)	• Treatment and diagnosis of children with ASD
**2**	**Pediatrist (*n*** **= 6)**	• Male (*n* = 6)	• Pediatrics (*n* = 4) • Pediatrics neurology (*n* = 2)	• Faculty member (*n* = 4) • Head of the children hospital/clinic (*n* = 2)	• Treatment of children with ASD
**3**	**Therapist (*n*** **= 4)**	• Male (*n* = 4)	• Speech therapy (*n* = 2) • Occupation therapy (*n* = 2)	• Faculty member (*n* = 4)	• Treatment of children with ASD
**4**	**Administrator (*n*** **= 8)**	• Male (*n* = 6) • Female (*n* = 2)	• Health services management (*n* = 2) • General physician (*n* = 2) • Psychology (*n* = 3) • Nursing (*n* = 1)	• Faculty member (*n* = 2) • Head of a rehabilitation and training center for autism (*n* = 4) • Top manager in province primary health center (*n* = 2) • Top manager in state welfare organization (*n* = 2)	• Administration of ASD services
**5**	**Researcher (***n* **= 4)**	• Male (*n* = 2) • Female (*n* = 1)	• Cognitive neuroscience (*n* = 1) • Epidemiology (n = 2)	• Faculty member (*n*= 3)	• Research on ASD

^a^ n = number of participants

^b^position is related to the participants’ position or job experience from the past until the time of interview

### Participants’ viewpoints about implementation of ASD screening programs

Viewpoints of the participants about ASD screening program are categorized in two main themes included screening children with early signs of ASD and screening all children in a specific age range (see Table 4).

**Table 4:** Participants’ viewpoints about implementation of ASD screening programs

Main Themes	Sub-Themes	Quotes
1. **Screening children with early signs of ASD**	• Impose lower cost to health system • Voluntary participation of families	• “...Furthermore, passive screening method (Screening the children who have revealed early signs of ASD) has lower cost than active ones (the universal screening). I think, there is no need to screen everyone, therefor, this method of screening (the universal screening) imposes less cost to health system. However, there is a vital need to inform public on signs/symptoms of the disorder.” (Psychiatrist) • “Although we expect to achieve more accurate results while screening based on parents’ concerns compared to the universal screening, in some cases parental over-concern creates bias in diagnosis process.” (Psychiatrist)
2. **Screening all children in a specific age range**	• Highlighting the importance of providing ASD services in public sector • As a right for every child • Require necessary infrastructure	• “Current services for children with ASD, particularly in public sector, are not adequate. One reason is that the policy makers do not consider providing these services as a priority. We know the prevalence of this disorder is high among children. It is necessary to understand that children are our country’s future human capital. The universal screening would highlight the importance of providing ASD services in public sector.” (Pediatrist) • “Having access to the universal screening is a right for every child. If I knew that my daughter’s specific behavior was due to her especial condition (ASD), I would have understood her condition earlier, and I would have tried to find a better way to communicate with her.” (Parent 5) • “If the aim of the implementation of ASD screening is solely providing statistics, then the screening would provide some vague results. It would not have a lot of benefits. The greater attention to statistics in recent years has led to an over-diagnosis of autism, while we faced under-diagnosis about 4 to 5 years ago.” (Pediatrist)

#### Theme 1: screening children with early signs of ASD

This theme summarizes the pros and cons of screening children with early signs of ASD. In such screening program, parents’ concern regarding their child’s health status is a base for implementing ASD screening.

#### Sub-theme 1–1: impose lower cost to health system

Professionals declared that screening children after observing the signs of ASD has lower operation costs than the universal screening program.

#### Sub-theme 1–2: voluntary participation of families

Psychiatrists had different viewpoints on accuracy of parents’ responses in screening children with early signs of ASD. Some psychiatrists believed that greater accuracy of responses is an advantage of screening children based on early signs of ASD compared to the universal screening program, because of the voluntary participation in the program and high motivation of participants.

*“When we do screening based on parents’ concern about their child’s abnormal behavior, we achieve more valid responses. (Psychiatrist).*

In contrast, some other psychiatrists believed that such screening method may create some biases in the results due to over-report or under-report of symptoms expressed by the parents of children with abnormal behaviors.

#### Theme 2: screening all children in a specific age range

This theme explains advantages and disadvantages of the universal screening, in which all children in a certain age are screened for ASD.

#### Sub-theme 2–1: highlighting the importance of providing ASD services in public sector

All types of participants both parents and professionals believed that universal screening has some benefits for children with ASD. For instance, universal screening would contribute to determine the prevalence of ASD in the community and it would encourage policy makers to provide necessary treatment for children with ASD.

#### Sub-theme 2–2: as a right for every child

All the parents emphasized that the universal screening program should be implemented during the routine child-health visits in PHC centers. They believed that the early diagnosis of the disorder would provide a good opportunity for them to seek the best available treatment in the early stages of the disorder.

*“I was seeing my child having difficulties in doing some activities and unusual repetitive habits at the early age, but I didn’t know what it meant precisely! If the healthcare provider had informed me of my child’s disorder in child-care visits, my son’s health status could have been better by now.” (Parent 7).*

All the parents believed that the implementation of a universal screening program is inevitable despite these children’s other issues like access to health services, quality of services, and effectiveness of services.

*“Having access to the universal screening is a right for every child. If I knew that my daughter’s specific behavior was due to her especial condition (ASD), I would have understood her condition earlier, and I would have tried to find a better way to communicate with her.” (Parent 5).*

#### Sub-theme 2–3: require necessary infrastructure

Although most participants from health system had positive opinion regarding the screening per se, they did not agree to implement universal screening program due to shortages of financial and human resources for providing appropriate rehabilitation services for children with ASD. Every participant had different rationale why it is not an appropriate time to implement the universal screening program. However, there was a consensus among the participants about the implementation of screening when parents reach out them with any concern about their children’s abnormal behaviors.

*“In my opinion, screening should be conducted based on parents’ concerns regarding their children’s condition. I don’t agree with the universal screening in current circumstances.” (Therapist).*

Moreover, most of the participants from health system agreed that the implementation of the universal screening program by itself does not create any value. In fact, the universal screening program is valuable if it is conducted following by early treatment and rehabilitation services.

### Challenges in implementation of the universal screening program

Entirely, four main themes including: shortcomings of screening tools, weakness of the health system, lack of coordination among ASD service providers, and social and ethical issues, sub-grouped to 11 sub-themes, were extracted to point out the challenges of the implementation of the universal screening program in Iran (see Table 5).

**Table 5:** Challenges in implementation of the universal screening program in Iran

Main Theme	Sub-Theme	Quotes
3. **Shortcomings of screening tools**	• Lack of reliable screening tool • Judgmental bias and lack of valid diagnostic assessment	• “I always use M-CHAT but I don’t get correct results. There is an essential need for cultural adaptation of screening tools (for ASD) in our region.” (Psychiatrist) • “Autism screening is very similar to using ASQ for developmental screening. ASQ is the same. For example, when we use ASQ for 1-year-old children, we expect to find 10–12% failed results, but our failures are much less than expected rate! It is approximately 1%!” (Psychiatrist)
4. **Weakness of the health system**	• Shortages of healthcare providers in the field of ASD • Lack of standard treatment protocols • Deficiencies in qualified screeners for ASD	• “We definitely cannot say that the improvement of a child’s health condition is related to the services provided for him/her, or is related to the cycle of disease itself. A population-based screening program (universal screening program) could not be the best option while these issues remain unaddressed.” (Pediatrist) • “Currently, there are very few therapists who are following the standards of treatment. First, we need to make sure that healthcare providers follow treatment standards, and then we can think about the implementation of screening.” (Psychiatrist) • “Screening is a main focus of the district health centers, and we have no qualified staff in our health centers.” (Administrator)
5. **Lack of coordination among service providers**	• Lack of integrated system for ASD • Problem of referrals	• “...Many children come to my clinic with several unnecessary medical test results including MRI, metabolic tests, and so on.” (Pediatrist) • “The referral system of autism is not performing well. For example, I referred a child with autism to his hometown, nevertheless, after some time, I realized that no service had been provided for him, and nobody had followed the referred child. These problems would be intensified after the implementation of the universal screening program.” (Psychiatrist)
6. **Social and ethical issues**	• People’s negative attitudes towards the autism • Unawareness of community about ASD • Uncertainty about the competence of autism centers • Lack of strong rational to implement the universal screening program	• “In one case, after discussing with me (as a psychiatrist), the Behvarz contacted the father of the child and told him about probability of his son’s autism disorder. The father became so angry and used offensive language against the Behvarz. Because the father was thinking his son was healthy and she (Behvarz) was labelling his son wrongly! In my opinion, the universal screening program should not be implemented till the adequate awareness and information being provided in the society about autism.” (Psychiatrist) • “Most people do not want to accept their child’s illness. It is very difficult to convince a parent to admit that his/her child has autism. For instance, I know a man whose son has autism too. When I started to talk about our children’s condition as a same problem, he slipped away. He said that my son doesn’t have autism, he is only a few hyperactive!” (Parent 6) • Most of the autism rehabilitation centers are private organizations or are affiliated to state welfare organization. We don’t know how these centers perform well in provision of services to children with ASD! (Administrator) • “The diagnosis of the disorder by itself cannot encourage families to seek an appropriate treatment. Currently, only few numbers of children with ASD have access to appropriate health care. How many of the families can afford their children’s treatment?! Screening is not ethical without having therapeutic and educational interventions for children who would be diagnosed with ASD.” (Therapist)

#### Theme 3: shortcomings of screening tools

This theme highlights the problems regarding the screening tools in Iran. ASD screening tools are instruments designed to help identify children with ASD. Screening tools could be specified for being used in medical centers, schools, or in other settings. Screening tools do not conclusively result in diagnosis, and a follow-up assessment should be conducted in each positive screening result.

#### Sub-theme 3–1: lack of reliable screening tools

Most of the participants, especially psychiatrists and therapists, agreed that the commonly used screening tools (M-CHAT and Q-CHAT) do not satisfy users in Iran. Participants with higher experience in ASD diagnosis declared that they use M-CHAT, however, they cannot reach accurate results in most of the cases.

*“Commonly used screening tools are not adapted in Azeri or other local languages (in Iran). This makes it difficult to use them appropriately with accurate results.” (Researcher).*

#### Sub-theme 3–2: judgmental bias and lack of valid diagnostic assessment

Psychiatrists and therapists declared that screening and diagnosis process differs from one health provider to another; because, they use different screening tools. Even when two clinicians use the same tool (M-Chat for instance), they usually obtain different results for the same case. On the other hand, the result of screening procedure entirely depends on screener and parent’s subjective judgment. Therefore, reliable results are not obtained in most cases.

*“Screening should be done by very precise criteria; however, case (child with ASD) detection depends extremely on individual judgments.” (Therapist).*

Another important challenge in screening is related to the large number of false-positive and false-negative results. Because of the judgmental nature of ASD diagnosis, the rate of false-positive and false-negative results are high. This issue highlights the importance of diagnostic assessment and tools, which interrupts the connection between screening and treatment consequently.

“*… We need to conduct more researches in terms of the development of a reliable diagnostic tool.” (Psychiatrist).*

#### Theme 4: weakness of the health system

This theme refers to the concerns about therapeutic system as one of the most important challenges in ASD screening program. This theme demonstrates that health system is not ready to provide effective and appropriate services for children with ASD.

#### Sub-theme 4–1: shortages of healthcare providers in the field of ASD

Participants from health system were critical regarding the large volume of patients in autism centers. ASD services, especially speech and occupational therapy, are pretty time consuming. Obviously, the autism centers do not have enough human resources to provide services for all children with ASD.

*“The number of therapists who provide rehabilitation and treatment services for children with ASD are limited. We currently suffer from limited resources*, *both financial and human resources. If the universal screening program is implemented more children with ASD is detected, therefore, such problems are exacerbated.” (Psychiatrist).*

All specialists with experience in the treatment of children with ASD had consensus that one major problem in providing services for these children is shortcomings of skills and knowledge among healthcare providers. Currently, most healthcare providers are beginners and treatment of children with ASD is their first work experience. Additionally, there is no specific course about ASD in their university education period.

#### Sub-theme 4–2: lack of standard treatment protocols

All participants from health system argued that one of the most important problems in provision of ASD services is related to lack of standard treatment protocols. They emphasized that implementation of universal screening program requires having the standard protocols to diagnose, treat, educate, and empower the children with ASD.

#### Sub-theme 4–3: deficiencies in qualified screeners for ASD

This theme indicates the concerns about the screeners’ low capability as one of the main challenges in the process of the universal screening program, particularly in rural areas. Screening programs require the screeners to have formal training about screening tools, content of questions, and the way questions should be asked. In Iran, primary health system is responsible for planning and conducting screening programs. In rural areas, Behvarzes (rural health-workers), who have a low-level of educations- mostly elementary degree, should conduct the screening, and this is an issue raised by the professionals regarding the capability of Behvarzes to implement the screening accurately.

*“… Screening is a specialized procedure, and it needs special skills. Behvarzes with low-level of education would not be able to perform it correctly, even after passing the special training courses!” (Therapist).*

Most of the participants from health system argued that community health workers either in rural or urban areas are not qualified to implement the screening. In fact, they believed that Behvarzes and other community health workers not only do not have an experience of treating or dealing with children with ASD, but also, they have not taken theoretical and practical courses in the fields of ASD.

#### Theme 5: lack of coordination among service providers

This theme remarks the concerns of the professionals regarding the problems in stratification of services as a challenge in ASD screening program. Currently, the stratification of services is not clear in terms of ASD services in public health system in Iran.

#### Sub-theme 5–1: lack of integrated system for ASD

Professionals declared that provision of services for children with ASD is completely vague in Iran’s health system. Such services are not provided in primary health system, and because of the specific and unknown characteristics of ASD, most of the parents do not know where to seek the diagnosis or treatment services for their children with abnormal behavior.

*‘Some of our colleagues say that they magically treat autism! Because of that, some families from rural areas, where their home, relatives and source of income are located, come to Tabriz (the capital of East-Azerbaijan province) to receive those miraculous treatments. However, wasting a lot of time, they do not see any significant improvement (in their child’s condition) after receiving the services. Therefore, these families realize that they have lost everything! If there was a stratification of services, this would have not been happened for such families.” (Psychiatrist).*

#### Sub-theme 5–2: problem of referrals

Furthermore, professionals believed that referral system is not efficient for ASD. Referrals are carried out without any specific criteria, getting routine feedback, and proper control by a superior.

#### Theme 6: social and ethical issues

This theme describes the challenges regarding the community’s attitudes towards autism and ethical issues that should be taken into consideration before the implementation of the universal screening program.

#### Sub-theme 6–1: People’s negative attitudes towards the autism

One of the most important issues highlighted by the participants from health system is related to cultural environment. They thought that people in Iran do not have enough information about the autism; therefore, their reaction is unpredictable after hearing about their child’s condition.

#### Sub-theme 6–2: unawareness of community about ASD

Parents of children with ASD expressed that most parents do not know anything about autism. They do not want to accept that their child has ASD; instead, they prefer to believe that their child is relatively hyperactive.

*“When my child was diagnosed with autism, I had not heard anything about autism. I had no idea about that. As a result, I did not start looking for any appropriate treatment at the beginning stages of the disorder.” (Parent 11).*

#### Sub-theme 6–3: uncertainty about the competence of autism centers

Autism services are mainly provided by private sector and rehabilitation centers affiliated to state welfare organization; however, health system has no authority to evaluate the competence of these centers. According to professionals, implementation of the universal screening will be unethical if the issues related to competence of centers are not approved.

“There is an ethical issue. We assume that the universal screening program is easy to implement. We think that the process of screening starts by diagnosing the disorder and continues well by referring a child with ASD to an autism center. But we do not know the answer of some serious questions; for example, are these autism centers able to meet the children’ needs? And, can we measure the competency of these centers?” (Psychiatrist).

#### Sub-theme 6–4: lack of strong rational to implement the universal screening program

All the participants from health system agreed that the implementation of the universal screening is not ethically correct without having a plan for providing accessible and effective treatment for any child would be diagnosed with ASD, and a reliable and valid screening tool.

*“.. Implementation of the universal screening program for only providing some numbers (statistics) is unethical!” (Pediatrist).*

### Participants’ recommendations to overcome the challenges

After synthesizing the data, three themes and seven sub-themes were extracted explaining the participants’ recommendations for better implementation of ASD screening and overcoming the aforementioned challenges. Main themes include development of supportive programs, improvement of ASD services, to provide better coordination between policy making, resource allocation, and service provision. (see Table 6).

**Table 6:** Participants’ recommendations to overcome the challenges

Main Theme	Sub-Theme	Quotes
7. **Development of supportive Programs**	• Raising community awareness • Empowering the parents of children with ASD • Development of support groups	• “Television interview with parents of children with ASD could be an effective way to increase the awareness of people.” (Parent 8) • “Parents of children with ASD are the most important part of the care. They should be completely aware about their child’s condition, treatment process, and consequences of the interventions. The parents would have active roles in their child’s treatment process; therefore, a great amount of attention needs to be paid to parent empowerment programs.” (Therapist) • “We have a critical role in implementation of the universal screening. We can share our own experience with parents of children who have newly been diagnosed with ASD.” (Parent 1)
8. **Improvement of ASD services**	• To develop setting/culture-based educational package • To provide special training for professionals in the field of ASD	• “To progress in the screening, we should indicate which child needs which treatment package.” (Psychiatrist) • “Before implementation of the screening program, primary educations must be provided to screeners both theoretically and practically about the process of the screening, screening tools, and the content of screening questions.” (Psychiatrist)
9. **To provide better coordination between policy making, resource allocation, and service provision.**	• Making a close collaboration of stakeholders • To implement applied researches	• “If donors provide funds for vital needs, resource allocation problem could be solved. Therefore, donors, managers, and therapists should work closely with each other.” (Administrator) • “One of our most important challenges in implementation of ASD screening program is the lack of knowledge about population’s attitudes and ideas regarding to autism. Therefore, we need to conduct more qualitative studies to understand their knowledge and attitudes about autism.” (Pediatrist)

#### Theme 7: development of supportive programs

This theme illustrates that the support programs are needed to help parents of children with ASD to better understand their child’s specific condition and seek the available services (s) as early as possible.

#### Sub-theme 7–1: raising community awareness

Approximately all participants both parents and professionals had consensus that there should be a unique program to enhance the awareness of people about autism and its consequences, and to introduce new and different ways of diagnosis and treatment. Awareness increasing campaigns could be implemented through mass media, street banners, mobile apps, or in the form of community education programs in PHC centers.

*“People’s knowledge about autism is not enough, and in most of the cases, their knowledge is not correct. Awareness regarding autism must be increased before the implementation of the screening program. The government can do this task through television shows, social network apps, banner, and so on.” (Therapist).*

#### Sub-theme 7–2: empowering the parents of children with ASD

All the participants, both professionals and parents, suggested that parents of children with ASD need to be empowered to take care of their children. Therefore, a special program is needed for empowering the parents of newly diagnosed children with ASD.

#### Sub-theme 7–3: development of support groups

There was an agreement among the parents regarding the development of support groups to help the parents of newly diagnosed children with ASD to adapt themselves with new condition. In such groups, older members would help new ones to manage their life and understand their child’s disorder by sharing their own experiences. Thus, such groups could facilitate the acceptance of the new situation for the parents and would motivate them to search for available services.

*“I think parents of children with ASD should form a group and provide advices for the new ones. They may be better in convincing the new diagnosed children’s parents regarding their children’s disorder compared to health care providers.” (Parent 10).*

#### Theme 8: improvement of ASD services

This theme describes the recommendations of the representatives of health system for better implementation of ASD universal screening program. In summary, the better results of the universal screening program will be achieved if desired services are accessible for every newly diagnosed child with ASD.

#### Sub-theme 8–1: provision of setting/culture-based educational packages

Researchers and psychiatrists believed that preparation of age-adjusted treatment protocols could be useful tools to manage the high volume of patient flows. Such packages should be prepared differently for each severity of spectrum and also, they should be designed separately for the target users such as parents, therapists, etc.

“Before beginning the screening program, we should develop appropriate treatment protocols based on our community’s culture. We can prepare several packages that are specified for health center, school, and home. These packages could reduce the burden for the therapists.” (Researcher).

#### Sub-theme 8–2: to provide special training for professionals in the field of ASD

Psychiatrists suggested that training courses should be provided for all screeners such as Behvarzes, PHC providers, and therapists before initiating the screening program. Training courses should have both theoretical and practical materials. In addition, trainees should be evaluated at the end of training courses. Those trainees who pass the courses should get license that shows their eligibility and credibility for conducting the screening.

#### Theme 9: to provide better coordination between policy making, resource allocation, and service provision

This theme shows that how coordination of policy making, resource allocation, and service provision could contribute to overcome challenges in implementation of ASD screening program in Iran.

#### Sub-theme 9–1: making a close collaboration of stakeholders

According to the participants from health system, many of the challenges will be resolved if close coordination is made among policy makers, donors, service providers, and specialists.

*“We need integration among healthcare providers, decision makers, trainers, and donors. If there was an appropriate coordination among all stakeholders, waste of resources would have been reduced.” (Researcher).*

According to administrators, if there is an effective collaboration between donors and policy makers in top level of the system, resources will be allocated to the most needed areas.

#### Sub-theme 9–2: to implement applied researches

Professionals suggested that efficient and effective implementation of the universal screening program requires conducting comprehensive researches to provide an appropriate and valid screening and diagnostic tools, educational packages based on specific needs of school, home, medical center, and other community settings, context-oriented treatment protocols, etc. It seems that it is very difficult to achieve these goals without having a specialized professional autism research center.

*“… We need to conduct more researches about the development of a reliable diagnostic tool, context-based treatment protocols, etc. To achieve these goals, it is necessary to have a professional autism research center.” (Psychiatrist).*

Additionally, administrators recommended that it would be worthy to implement the universal screening program in a few selected health centers to conduct economic evaluations.

*“We should implement the program (the universal screening) in four small health centers in Tabriz to evaluate its cost-effectiveness; because, the health system suffers from limited resources in rural areas, and, implementation of the screening program can’t be accomplished if it is not cost-effective.” (Administrator).*

Most of the professionals suggested several qualitative studies to investigate the population’s viewpoints, knowledge, and attitudes about ASD.

## Discussion

This qualitative study, which is a part of Azeri Blue Buddies: Interdisciplinary Longitudinal Autism Researches (ABBILAR), is the first to investigate the viewpoints of different types of stakeholders about the implementation of ASD screening program in East-Azerbaijan, Iran. All participants believed that early treatment for children with ASD requires early diagnosis. However, the early diagnosis does not guarantee the early treatment. According to the participants from health system, Iran’s health system is not currently ready for the implementation of the universal screening program because of lacking necessary resources (e.g. financial, professional, time, etc.) and cultural barriers.

The specialists and therapists recommended that the screening of children should be implemented based on parents’ concerns raised about their child’s condition rather than conducted for all children. A qualitative study by Fenikilé et al. (2015) found the same result [27]. In our study, parents agreed that the implementation of universal screening program is necessary, and it should be integrated into primary child-care visits in early ages of children. However, recommendations about the implementation of ASD screening program or other developmental disorders are not consistent in literature. For instance, the American Academy of Pediatrics recommended using standardized screening tools in routine well-child care [20]. In contrast, according to the National Screening Committee in UK (2012), the screening of ASD was not recommended for all children [28]. In current study, the discrepancy between parent and professional viewpoints may be due to differences in their expectations from the health system as well as differences in their interests, priorities, and experience. Based on Carbone et al. findings, similar discrepancy between the parent and professional viewpoints, and dilemma between having an early diagnosis and not having adequate treatment resources, is a common challenge even in developed countries like the US [29].

According to the participants, implementation of universal screening program for ASD faces several challenges. One of the most important challenges is judgmental bias. Professionals declared that there have been many false-positive and false-negative biases in diagnosis of ASD in Iran. Such biases will be increased when the screening is conducted by non-professional and low-educated staff. Furthermore, in East-Azerbaijan, although most of the professionals use M-CHAT, they are not satisfied with that. A study by Al-Qabandi et al. (2011) showed that because of the similarity of the diagnosis of autism to several neural and developmental diseases like cerebral palsy and neurodevelopmental disability, various features of diagnostic tools may give different results for the same person evaluated by two clinicians using different tools [11]. Results of a qualitative study by Crais et al. (2014) confirmed that access to an effective screening tool is one of the main elements to successfully implement the ASD screening program [30]. Evidences regarding the use of common screening tools such as M-CHAT are mixed [31, 32]. For instance, a study showed that M-CHAT did not diagnose all cases and some children were diagnosed with ASD later [33]. On the other hand, several other studies showed reversed results [9, 18]. Professionals in East-Azerbaijan do not obtain similar outcomes using common diagnosis tools. One possible reason could be that these tools have not been validated in Azeri language. According to variability of psychiatric signs in terms of the language properties, this issue may be problematic. There is an essential need for contextual adaptation of ASD screening tools in Azeri language, in Azeri region of Iran. Results of a few systematic reviews corroborates that cultural adaptation of the screening tools is essential for a ASD screening program and could improve the results of ASD screening in LIMICs [21, 34]. For appropriate implementation of the universal screening program, an specific screening tool for ASD should be designed for preschool, primary school, PHC setting, and household [35]. To avoid inappropriate labelling of the child, there is an essential need to reduce false-positive results as much as possible. This cannot happen until valid and reliable screening tools are developed.

another challenge of ASD screening in East-Azerbaijan province was related to treatment issues. In Iran, main interventions for children with ASD include speech therapy and occupational therapy, which are mainly provided by speech therapist and occupational therapist with BSc, MSc, or Ph.D. degrees. All the rehabilitation interventions are provided in private autism and disability rehabilitation centers with no insurance coverage. The only financial coverage is a subsidy paid by state welfare organization for its affiliated autism rehabilitation centers. The subsidy covers a small portion of the rehabilitation services costs. Some children with ASD go to regular schools; some others, who are more severely affected with ASD, go to autism and intellectual disability private schools. Some parents as well as some therapists believed that current treatments are effective in improving children’s behavioral conditions. However, the majority of the professionals believed that process of rehabilitation therapy does not fit the standards. Issues related to the treatment system were highlighted as an important challenge which should be solved before initiating the universal screening program. In line with our results, Al-Qabandi et al. reported that due to lack of curative outcomes in therapeutic procedures, and high financial burden of the treatment, there was not enough support for the implementation of a universal screening program for autism [11]. According to the study of Robins, appropriate and early treatment should be available to have a successful and effective screening program for ASD [18]. Hobart HM investigated that delay in early intervention is one of the most serious problems for children with ASD, from the parents’ perspective in China [36]. Mandell & Mandy’s editorial paper (2015) highlighted that the universal screening program should be implemented in a country with available treatments for all young children diagnosed with ASD [37]. All these findings corroborate our results.

Another challenge of the universal screening for ASD, that both parents and professionals mentioned as a major barrier to both diagnosis and treatment of children with ASD, is social stigma. Social stigma results in delay in diagnosis and inadequate access to services. Several studies in line with our results have shown that cultural factors and social stigma delay timely autism diagnosis and treatment [38, 39]. Although evidence shows that families of children from minority racial and ethnic communities are particularly vulnerable to stigma [38, 40], a study has shown that families of higher socioeconomic status and White race were more likely to refuse to participate in a screening program for ASD due to social stigma [31]. Social stigma is what most families of a child with ASD have experiences in their life. Nevertheless, authorities need to take this into consideration when planning for ASD screening by increasing the awareness of the community about ASD as well as considering special family empowerment programs for families of children with ASD to cope with this challenge.

The results of current study indicate that early treatment requires early diagnosis. Nevertheless, early screening does not guarantee the early diagnosis in the absence of valid and reliable screening tools and methods. In spite of our assumption that early diagnosis may improve the treatment, there is no study to support this assumption. To our knowledge, there is no study showing that a screening program has influence on process, incidence, and the accuracy of ASD diagnosis, particularly in Iran. Thomaidis and colleagues (2015) found that screening at earlier ages may increase false-positive diagnosis [41]. Nonetheless, in our study, the parents suggested that the universal screening program should be conducted in routine child-care visits irrespective of the system and policy challenges. The parents believed that they need to be aware of their child’s condition as soon as possible; because, this would provide an opportunity for them to seek available treatment(s), and, could help them to have better communication with their child. The parents had consensus that development of a support group would help the parents of newly diagnosed children with ASD to come up with their child’s disorder.

### Implications of the study

The results of this study could be used in making a decision regarding the implementation of a screening program not only in Iran but also in other LMICs countries with similar context. Many LMICs face similar challenges in the provision of services for children with ASD. Social stigma, which was remarkably highlighted as a major barrier to diagnosis and treatment, is a common challenge that families in even high-income countries face. Results of this study will provide policymakers with crucial information on the requirements of the implementation of the universal screening for ASD to adopt appropriate strategies to overcome these challenges.

### Limitations

We acknowledge the limitations of this study, that the participants were only from a province of Iran. However, East-Azerbaijan is the sixth most populated province among 31 provinces and has 3.8 million population that 28% live in rural areas similar to the national proportion (26% of the population of Iran live in rural area). Also, several children with autism refer to Tabriz (capital of East-Azerbaijan) from neighboring provinces. The second limitation is that we didn’t recruit the parents of children without ASD because according to the meetings we had with research team members to plan the study, parents of children without ASD do not have good knowledge on the ASD and could not have an in-depth understanding of the topic.

## Conclusion

Professionals believed that ASD universal screening program is valuable if it leads to an early intervention. They stated that it is not possible to provide appropriate and effective services to all children with ASD after implementation of the universal screening program. In fact, according to professionals, there is not enough rationale to implement ASD screening program for all children. The financial burden of the treatment, lack of resources to support the newly diagnosed cases, lack of appropriate screening tools, lack of standard treatment protocols, lack of qualified professionals, lack of educational packages to be used in treatment centers/home/school, lack of knowledge regarding the attitudes of people about autism, and lack of authority to assess the competence of autism centers (where the children would be referred to) were identified as the most important challenges for implementation of screening program in short term. Other developing countries may have the same challenges. Moreover, parents of children with ASD believe that the implementation of universal screening is inevitable, and they expect that ASD screening program should be implemented in primary health centers, during child-care visits in early stages of children’s life. Parents and professionals had opposite views on ethical aspect of the implementation of ASD screening program. According to the results of this study, implementation of the screening program should be followed in steps; first, assurance of access to quality and effective services; second, building appropriate screening tools; third, increasing public awareness on ASD; fourth, developing the training programs for parents of children with ASD; fifth, training and employing necessary human resources, and finally implementing the screening. Inclusion of the representatives of health system along with parents of children with ASD improved transferability of the study findings. The results of this study open up unspoken issues that could help in initiating the screening program not only in Iran but also in other LMICs too.

## Supplementary Information

**Additional file 1.** Contextual information about the organizational structure of services for children with ASD.

**Additional file 2.** Questions asked from participants.

**Additional file 3.** Theme-trees supporting our results.

## Data Availability

Data used in this study are qualitative audio taped data scripted verbatim; therefore, according to confidentiality principles we cannot share original data. However, main data are reported in result section and in Additional file 3.

## Abbreviations

ASD: Autism spectrum disorders
ASQ: Ages and Stages Questionnaire
CAC: Comprehensive Autism Center
CHC: Comprehensive Health Centers
FGD: Focus Group Discussions
GP: General Practitioner
PHC: Primary health-care
USPSTF: US Preventive Services Task Force
