# Supplementary material for: Considerations about the implementation of an autism screening program in Iran from the viewpoints of professionals and parents: a qualitative study
Source: BMC Psychiatry. 2021 Jan 23;21:55. doi: 10.1186/s12888-021-03061-0 (PMC7825177; doi:10.1186/s12888-021-03061-0)
Supplement: Supplementary file 1 — Additional file 1. Contextual information about the organizational structure of services for children with ASD. [file 12888_2021_3061_MOESM1_ESM.pdf]

Additional File 1

Organizational structure of services for children with ASD in Iran

Ministry of Health is the main policy-maker body as well as health and medical care provider in Iran. Each university of medical science is the representative of the Ministry of Health in each province. The university is responsible for medical education as well as service provision in public facilities. The Ministry of Health has a nation-wide health network to provide and monitor public healthcare at district level. PHC is provided by Health House (in rural area) and Comprehensive Health Centers (CHC) (in urban area). There are two Behvarzes (one male and one female, usually married couples) in each Health House. Behvarzes are trained as community health workers, who are responsible for providing a range of PHC (from preventive to promotion) for newborns, pregnant women, children, elderly, etc. in rural areas. General physician visits are limited to a specific time when a general practitioner (GP) is available in the Health House. Behvarzes write down the names of persons who need to be visited by the GP, and call them to receive medical care when the GP is present in Health House. After visiting each patient, GP decides about referring he/she to a specialist or district hospital. On the other hand, each CHC has a full-time GP. In CHCs, healthcare providers provide health promotion and disease prevention services such as vaccination and screening for determined catchment area. In addition to a GP, there are other health care providers in CHCs such as dentists, dental hygienists, environment and occupation health professionals, and mental health professionals. All service providers in CHCs are required to have a bachelor’s or a master’s degrees in their field of expertise. Both Health House and CHC are responsive to district health center regarding the health status of their target population. Unfortunately, there are no diagnostic or therapeutics services for children with ASD in PHC. Currently, children with ASD could access diagnosis or treatment services if their parents take them to physicians' offices or professional behavioral disorder

centers such as CAC. A figure illustrating the position of CAC in health system is available in figure 1.

In Iran, the CAC is the first and only public center for children with ASD which is located in East-Azerbaijan, and provides 30% discount for each visit. In addition to patients from East-Azerbaijan, some children with ASD from the neighbor provinces are referred to this center. However, due to limited capacity of the center, the number of services provided for each child is to maximum 10 visits, and thereafter, children with ASD are referred to private centers. The state welfare organization is another public organization which subsidizes provision of services for eligible children with ASD in its affiliated autism or rehabilitation centers.

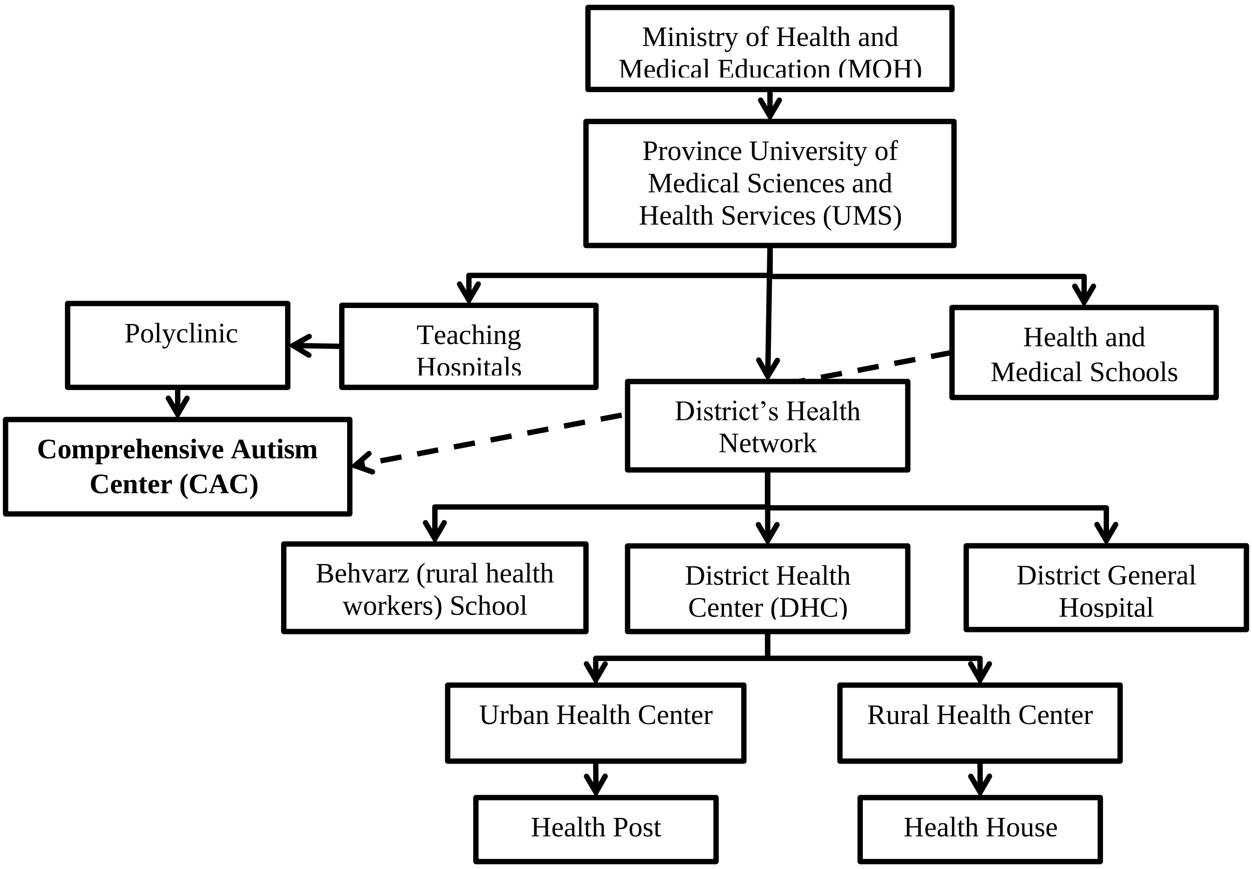

Fig. 1 Health system structure in Iran and Comprehensive Autism Center (CAC) position
