# Supplementary material for: Considerations about the implementation of an autism screening program in Iran from the viewpoints of professionals and parents: a qualitative study
Source: BMC Psychiatry. 2021 Jan 23;21:55. doi: 10.1186/s12888-021-03061-0 (PMC7825177; doi:10.1186/s12888-021-03061-0)
Supplement: Supplementary file 2 — Additional file 2. Questions asked from participants. [file 12888_2021_3061_MOESM2_ESM.pdf]

Additional File 2: Interview Protocol Form

Title: **Considerations about the Implementation of an Autism Screening Program in Iran from the Viewpoints of Professionals and Parents: A Qualitative Study**

Date ____________________________

Time ____________________________

Location _________________________

Interviewer _______________________

Interviewee _______________________

Participants' Characteristics
Gender: Female ☐ Male ☐
Age: ………………………..
Job: ……………………………
Years of Job Experiences: ……………………

Would you like to participate in this interview?
Informed consent was obtained from the study participant
Informed consent was NOT obtained from the study participant

Notes to interviewee:
Thank you for your participation. I believe your input will be valuable to this research and in helping grow all of our professional practice. Obviously, the information contained in the questionnaire is completely confidential. Participation in the survey is completely voluntary and will not affect you. We sincerely thank and appreciate the fact that you are helping the researchers with honesty and openness in commenting. Approximate length of interview: 30 minutes, five major questions.

Research questions:

1. What are your viewpoints regarding the ASD screening programs?
2. What are your viewpoints regarding the implementation of the ASD universal screening in Iran?
   - Benefits
   - Disadvantages
3. What challenges does an ASD universal screening face in Iran especially East-Azerbaijan?
4. What are your suggestions to overcome the challenges?
5. ***And so forth***
