# Supplementary figures and images for: Considerations about the implementation of an autism screening program in Iran from the viewpoints of professionals and parents: a qualitative study
Source: BMC Psychiatry. 2021 Jan 23;21:55. doi: 10.1186/s12888-021-03061-0 (PMC7825177; doi:10.1186/s12888-021-03061-0)

### Additional File 3: Theme-tree

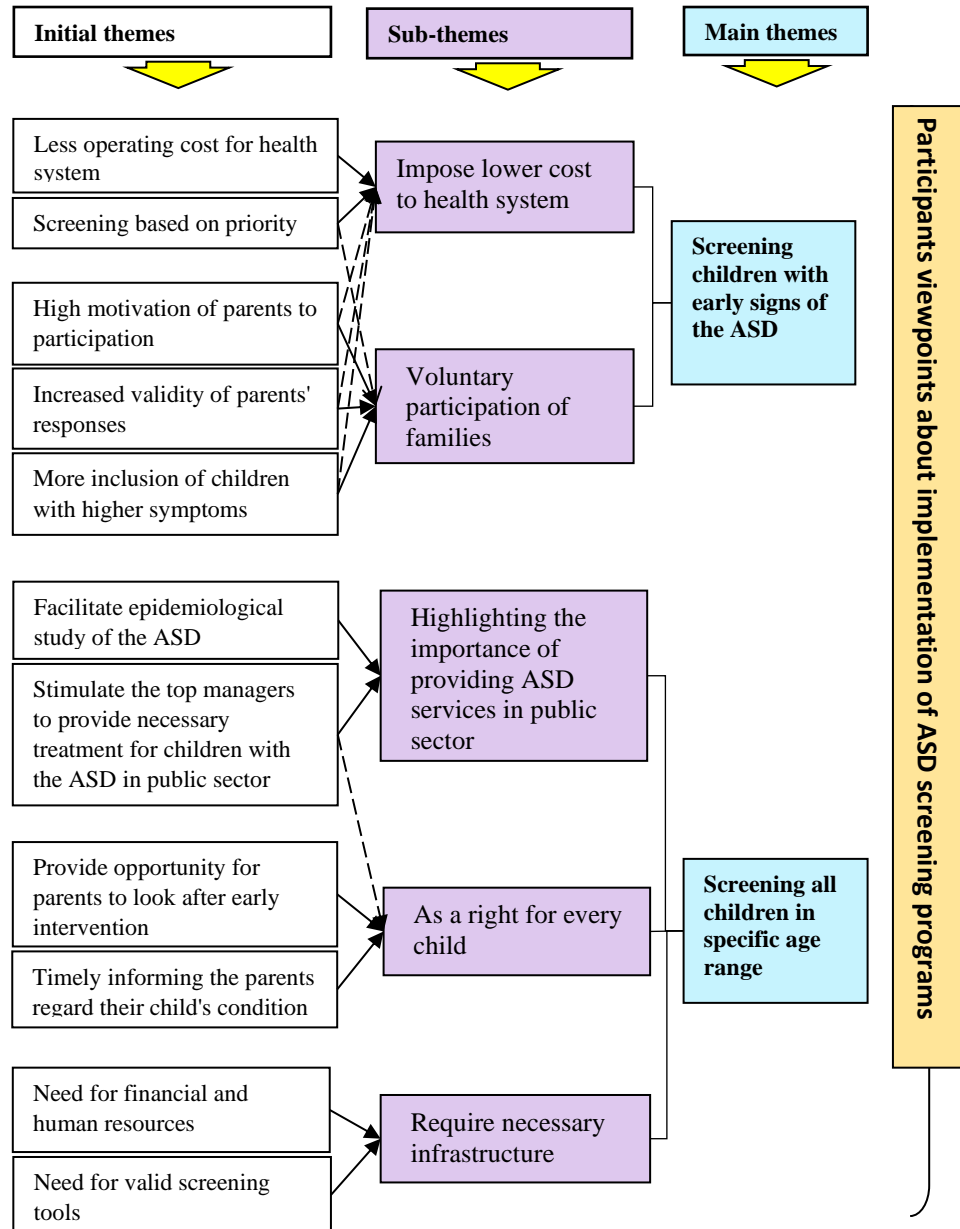

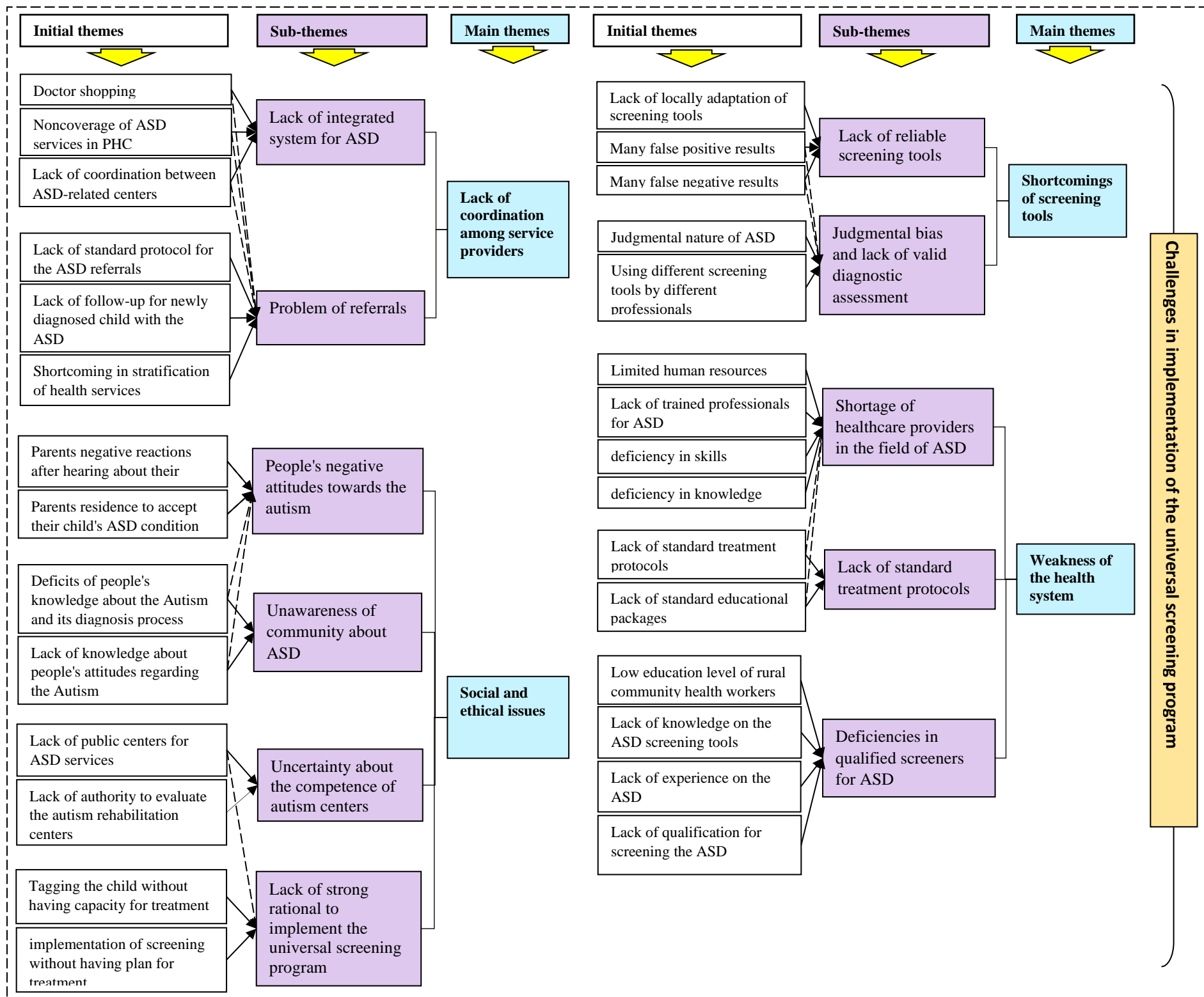

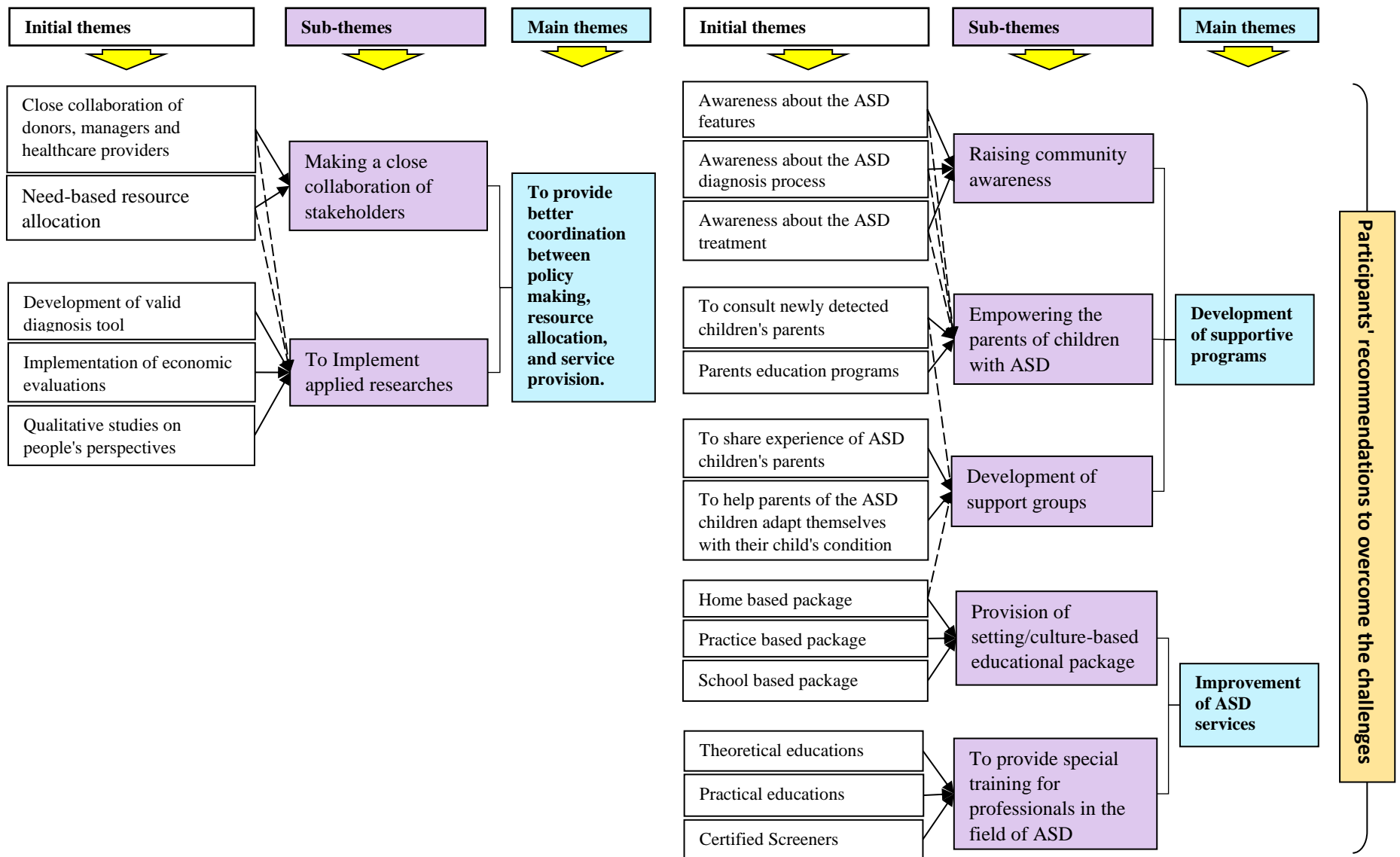

Supplement: Supplementary file 3 — Additional file 3. Theme-trees supporting our results. [file 12888_2021_3061_MOESM3_ESM.pdf]
